# Ethical challenges and evolving strategies in the integration of artificial intelligence into clinical practice

**DOI:** 10.1371/journal.pdig.0000810

**Published:** 2025-04-08

**Authors:** Ellison B. Weiner, Irene Dankwa-Mullan, William A. Nelson, Saeed Hassanpour

**Affiliations:** 1 Department of Biomedical Data Science, Geisel School of Medicine, Dartmouth College, Hanover, New Hampshire, United States of America; 2 Department of Health Policy and Management, Milken Institute School of Public Health, The George Washington University, Washington, DC, United States of America; 3 Dartmouth Institute for Health Policy and Clinical Practice, Geisel School of Medicine, Dartmouth College, Hanover, New Hampshire, United States of America; 4 Departments of Biomedical Data Science, Computer Science, and Epidemiology, Geisel School of Medicine, Dartmouth College, Hanover, New Hampshire, United States of America; National Tsing-Hua University: National Tsing Hua University, TAIWAN

## Abstract

Artificial intelligence (AI) has rapidly transformed various sectors, including healthcare, where it holds the potential to transform clinical practice and improve patient outcomes. However, its integration into medical settings brings significant ethical challenges that need careful consideration. This paper examines the current state of AI in healthcare, focusing on five critical ethical concerns: justice and fairness, transparency, patient consent and confidentiality, accountability, and patient-centered and equitable care. These concerns are particularly pressing as AI systems can perpetuate or even exacerbate existing biases, often resulting from non-representative datasets and opaque model development processes. The paper explores how bias, lack of transparency, and challenges in maintaining patient trust can undermine the effectiveness and fairness of AI applications in healthcare. In addition, we review existing frameworks for the regulation and deployment of AI, identifying gaps that limit the widespread adoption of these systems in a just and equitable manner. Our analysis provides recommendations to address these ethical challenges, emphasizing the need for fairness in algorithm design, transparency in model decision-making, and patient-centered approaches to consent and data privacy. By highlighting the importance of continuous ethical scrutiny and collaboration between AI developers, clinicians, and ethicists, we outline pathways for achieving more responsible and inclusive AI implementation in healthcare. These strategies, if adopted, could enhance both the clinical value of AI and the trustworthiness of AI systems among patients and healthcare professionals, ensuring that these technologies serve all populations equitably.

## Introduction and motivation

In the last decade, artificial intelligence (AI) has made remarkable progress, primarily due to advancements in deep neural networks, widely known as deep learning. The transformative impact of these methodologies in non-medical fields, such as robotics, autonomous driving, and speech understanding, has fueled interest in their application to medicine. As a result, AI models have been seriously considered for use in adjacent domains, such as biomedicine and healthcare.

To date, these promising technologies have led to the creation of sophisticated AI systems capable of performing critical clinical tasks, such as medical image interpretation at the level of expert physicians [1–4]. Some of these innovative AI technologies have been developed by our team at Dartmouth [5–12]. Until recently, creating AI systems to assist pathologists, radiologists, and other imaging professionals required laborious feature engineering—the manual design of algorithms to preprocess images, segment anatomic structures, detect features, and classify abnormalities. Developing these systems often took years, but recent advances in AI have merged feature engineering with deep learning from large sets of labeled or even unlabeled training data [13]. Deep learning approaches are highly adaptable to diverse imaging tasks. These AI systems have the potential to transform healthcare delivery due to their ability to analyze large datasets and recognize complex patterns, making patient care more efficient and accurate. This can reduce diagnostic errors and healthcare costs and, most importantly, improve patient outcomes.

Over the last decade, electronic health records have become the most comprehensive source of clinical information for biomedical research and decision-making due to widespread adoption. However, much of this information exists in free-text format within clinical notes and reports. The variability and ambiguity of unstructured free text create major obstacles to rapid extraction and reuse of clinical data. These records may also lack control and negative cases and often contain significant gaps, as seen when patients transfer between hospital systems, raising concerns about data completeness. Recent advancements in natural language processing (NLP), fueled by the exceptional performance of large language models across diverse tasks, have opened new avenues for AI in this domain [14–21]. These NLP methodologies can address the challenges of extracting information from unstructured data, enabling the development of informatics methods that unlock valuable insights for translational research and clinical care [22–24].

Undoubtedly, medicine is undergoing a rapid transformation driven by AI and machine learning, a trend that will continue at an unprecedented rate. As these changes unfold, the medical community faces pressing concerns regarding the ethical implementation of AI while legislation struggles to keep pace. AI ethics is a field focused on the responsible development, deployment, and use of AI within the constraints of current legal and ethical standards. The rapid pace of AI innovation demands an inclusive discussion among experts to ensure ethical use, prompting a surge of research in this area. No single group should dictate the solutions to these complex issues, nor should discussions become insular. Accordingly, this paper presents widely recognized challenges and proposed strategies for ensuring the ethical integration of AI into clinical practice. We will review the strengths of these recommendations and highlight areas that require further exploration.

## Core ethical challenges

In this section, we will present major ethical concerns associated with the integration of AI in clinical practice.

### Justice and fairness

#### Eliminate embedded bias in algorithms to ensure current bias is not exacerbated.

1. Justice and fairness in healthcare AI require equitable distribution of medical resources and unbiased decision-making. These principles encompass “distributive justice” (fair resource allocation) and “procedural justice” (fair decision-making) [25]. AI systems must avoid reinforcing biases that could disadvantage certain patient groups. Biases also intersect with Social Determinants of Health (SDOH), as algorithms trained on non-representative data can lead to unequal access, lower-quality care, and misdiagnosis in marginalized populations. Incorporating SDOH in data collection and algorithm development supports both distributive and procedural justice.
2. In a literature review of 45 sources, justice and fairness were the ethical issues of highest concern in 24 of the articles, arising in concert with themes such as bias, discrimination, and equality [26]. Strategies noted among the literature took both an algorithmic and data perspective, suggesting that developers purify algorithms of decision support tools, manage fairness constraints and distribution, guarantee responsible data collection, and encourage the cooperation of stakeholders in AI development.
  a. A widely used healthcare algorithm assessing overall health status assigned equal risk levels to Black and white patients, despite Black patients being significantly sicker. The algorithm used healthcare costs as a proxy for medical need, introducing implicit racial bias, as less is typically spent on Black patients. Adjusting for this disparity would increase care for Black patients from 17.7% to 46.5% [27].
3. Medical data typically reflects historical trends of discrimination through underrepresented minority groups in research and biased disease labels, thus mandating thoughtful design of AI to avoid exclusion. ChatGPT has been shown to exacerbate discriminatory biases by advising patients with identical symptoms, but distinct demographics differently [28]. Insured patients were advised to seek emergency care, but some uninsured patients were referred to community clinics.
4. Marginalized groups lack access and power to voice their concerns in AI decision-making or implementation. Unequal concentration of power and resource imbalance also raise concerns of justice, as only certain hospitals may have access to life-saving AI. Ethical solutions demand government support in the form of subsidization of AI for underfunded hospitals or regulatory frameworks designed to prioritize need, or open-source AI initiatives to increase access.

### Trustworthiness

#### Trustworthiness: Establish representative data to train and test an AI model while ensuring transparency [29].

1. Trustworthy AI systems in healthcare are grounded in transparency, explainability, and interpretability, as these qualities collectively ensure that models are safe, reliable, and fair across diverse patient populations. For healthcare providers to trust AI-based insights, they must be confident that these insights are generated from “representative data” that covers relevant demographic and clinical variations.
  a. One study examined the influence of multiple factors on patient trust of AI systems and devices in healthcare, finding that younger respondents (18–30) were more concerned with AI ease of use, accountability, risk, and social influence (their perception of AI being affected by those around them), while elder respondents (60+) were concerned with the practicality of AI and less sensitive to issues of risk and data privacy. The development of trustworthy AI should thus consider the expectations of all patients and include avenues for feedback and criticism during the process [30].
2. Machine learning is trapped in a circular paradox: AI applications have a “large need for data to keep learning and improving, hence becoming safer, hence more trustworthy” [31]. For models to be considered trustworthy, they must be well-trained with a representative dataset. Rare health conditions lack extensive amounts of data, thereby making the software less trustworthy in its results and explanations. However, big data can be worrisome in terms of reliability and complexity, thus reducing the model’s trustworthiness and, by extension, transparency.

#### Transparency: Address the problem of explainability within AI models by providing the ability to verify results and in-program qualities.

1. Transparency is a key principle of AI ethics, especially in healthcare, where trust and accountability are critical. It encompasses multiple dimensions: “data transparency” (clarity on data sources and representativeness), “algorithmic transparency” (insights into model structure and assumptions), “process transparency” (disclosure of development steps, including human interventions), and “outcome transparency” (explanation of how results are generated) [32]. Related concepts include “explainability,” the ability to describe how an AI model reaches conclusions, and “interpretability,” the extent to which humans can understand cause-and-effect relationships within a model. Together, these aspects help mitigate AI’s “black-box” nature.
2. The “black-box” problem in AI limits interpretability, making it difficult for developers to predict or explain model decisions due to complex internal architectures. This challenge is especially critical in healthcare, where solutions must be understandable to both caregivers and patients. AI-generated explanations are often inaccurate or misleading, as they are typically post hoc. Developers, healthcare organizations, and clinicians must ensure AI tools are trustworthy, user-friendly, and human-centric. Key considerations include sample size limitations, inappropriate statistical significance, and self-serving biases like “data shopping” [29]. These guidelines help address concerns related to data and process transparency.
3. Patients must know and understand the process behind healthcare decisions made by AI, and require care givers to explain to patients the limitations and reason for such AI-driven decisions [33].

### Patient consent and confidentiality

#### Acquire informed consent to use patient data and ensure anonymity.

1. Patient consent and confidentiality are fundamental ethical concerns in healthcare, especially as AI relies on large datasets. “Patient consent” upholds autonomy by allowing individuals control over their health data, while “confidentiality” prevents unauthorized access, fostering trust. AI presents unique challenges, as its need for diverse data can conflict with privacy rights. Without strong consent mechanisms and confidentiality protections, AI-driven healthcare risks violating privacy, undermining trust, and weakening ethical standards.
2. An inherent point of conflict exists in the decision of whether to prioritize comprehensive datasets for AI models, or guaranteeing consent of patients even if confidentiality is already ensured. Of course, data leaks are still possible thus preserving the importance of informed consent. In relation to issues of trustworthiness and transparency, increasing the rate of consent is an important area for improvement in the field.
3. A study on patient perspectives regarding informed consent in AI-driven diagnosis found that when physicians consult AI instead of human radiologists, patients place greater importance on AI use. This challenges the assumption that additional explanations are unnecessary in lower-risk or widely used settings or when AI outperforms physicians. Demographic factors such as gender, age, and income significantly influenced perceptions, highlighting the need for a personalized approach to disclosure [34].

#### Respect the privacy rights of users and third parties.

1. Patient trust and autonomy are affected by their right to privacy. Most “mobile disorder detection systems” risk data hacking as they use mobile devices to acquire signals, transfer, analyze, and forward the results to users in a stored database [26]. The same is true for online systems.

#### Remind patients of their ability to opt-out at any time and empower them to exercise autonomy in choice.

1. To respect patient autonomy, experts suggest clinicians discuss the topic in terms of trust, shared decision-making, and legal responsibilities of clinicians; a uniform understanding of issues involved in patient–clinician relationships; and ensure transparency regarding the use of AI to convey to patients that human-judgment takes priority over AI systems [26].
2. While consent may be acquired at the time of use, many AI models use this data in an ongoing fashion to continue updating. Thus, an additional challenge arises in considering whether patient consent should be frequently in discussion. Patients should have the right to opt-out at any time, but this may impact the model, which has already applied the data to learning algorithms.

### Accountability

#### Properly delegate and accept responsibility for transparent and ethical conduct.

1. Accountability is a key ethical concern in healthcare AI, defining responsibility for AI-driven decisions in patient care. Unlike traditional medicine, where clinicians are accountable, AI involves multiple stakeholders, including developers, providers, and institutions. Errors or unsafe recommendations complicate accountability, especially when AI systems are opaque or lack documentation. Without clear accountability, patient safety risks increase, and trust erodes. Robust frameworks are essential to ensure stakeholders prioritize ethical conduct and patient well-being.
2. Interactions between AI model developers, organizational leaders, and healthcare providers create risk in that they may not be inclined to take responsibility for errors. AI developers may fear monetary consequences over ethical considerations, while medical professionals may inadvertently place patients more at risk due to a subconscious feeling of immunity from the AI system. There is a clear misalignment of risk and return that requires ethical consideration.
3. Conflicting advice given by AI and medical experts will prove difficult for healthcare providers, as models often lack a measure of certainty. Further, the tendency of humans to default to decisions generated by machines as opposed to conflicting data (commission errors) or human decisions (omission errors, or the nonobservance of AI failures) is known as automation bias. This phenomenon involves blindly and earnestly accepting AI results, raising concerns of human culpability [35].
4. The role of organizations and healthcare institutions in terms of monitoring AI, assessing AI implementation, and oversight need to be included in this measure of accountability. Organizations promote certain regulations under which developers and providers are bound by. Another challenge relates to whether groups must disclose if they are using AI as part of shared decision-making.

### Patient-centered and equitable care

#### Ensure AI complements the role of primary caregivers.

1. AI can provide real-time insights, streamline diagnostic processes, and offer data-driven recommendations that assist caregivers in critical, complex situations [29]. By enhancing diagnostic accuracy and efficiency, AI allows caregivers to focus on the personal and emotional aspects of patient care, where human empathy and judgment are irreplaceable.
2. To align with the goals of patient-centered care, AI tools must be adaptable, ensuring recommendations are consistent with individual patient needs and preferences. This requires transparency in how AI systems generate insights, allowing clinicians to interpret AI outputs in a way that respects each patient’s unique context and circumstances.
3. Researchers comparing algorithmic and human diagnostic and treatment decisions found that patients are “more resistant” to the former due to concerns that the model can’t account for patient individuality, are less likely to use healthcare, and perceive negative utility. It is recommended that AI is personalized and deployed alongside a clinician to reduce resistance and the feeling of “uniqueness neglect” [36]. AI outputs should be treated as supportive tools rather than definitive instructions, empowering caregivers to make informed, context-aware choices that best serve their patients.

#### AI should communicate with empathy and equity to patients.

1. AI systems must be developed to support equitable healthcare delivery, which requires careful attention to socioeconomic, gender, and ethnic factors that can affect patient care. Research has shown that disparities in treatment, such as black women receiving less care when reporting pain [37], highlight the potential for AI systems to inadvertently perpetuate biases. Ensuring that AI is trained on representative data can help minimize biased responses and improve fairness across patient populations.
2. AI systems must also be designed to communicate in a way that respects patients’ emotional and psychological needs. This requires embedding a sense of empathy within AI interactions to create responses that are human-centered and sensitive to patients’ vulnerabilities. The goal is for AI to offer support in a manner that feels respectful and considerate, rather than impersonal or dismissive, fostering patient comfort and trust.
3. Finally, AI-driven insights and recommendations should provide clear, understandable explanations that help patients feel informed and reassured. By delivering transparent and granular explanations of diagnoses or treatment plans, AI can empower patients to make informed healthcare decisions and feel more involved in their care. This human-centric approach reinforces empathy and equity, helping to mitigate biases and promote fairness in healthcare.

## Emerging ideas

In the previous section, we covered the current concerns regarding the use of AI in healthcare applications. now we will discuss the potential frameworks that have been proposed to address these concerns and ensure ethical use of AI.

### Multiscale ethics

**Fig 1:**
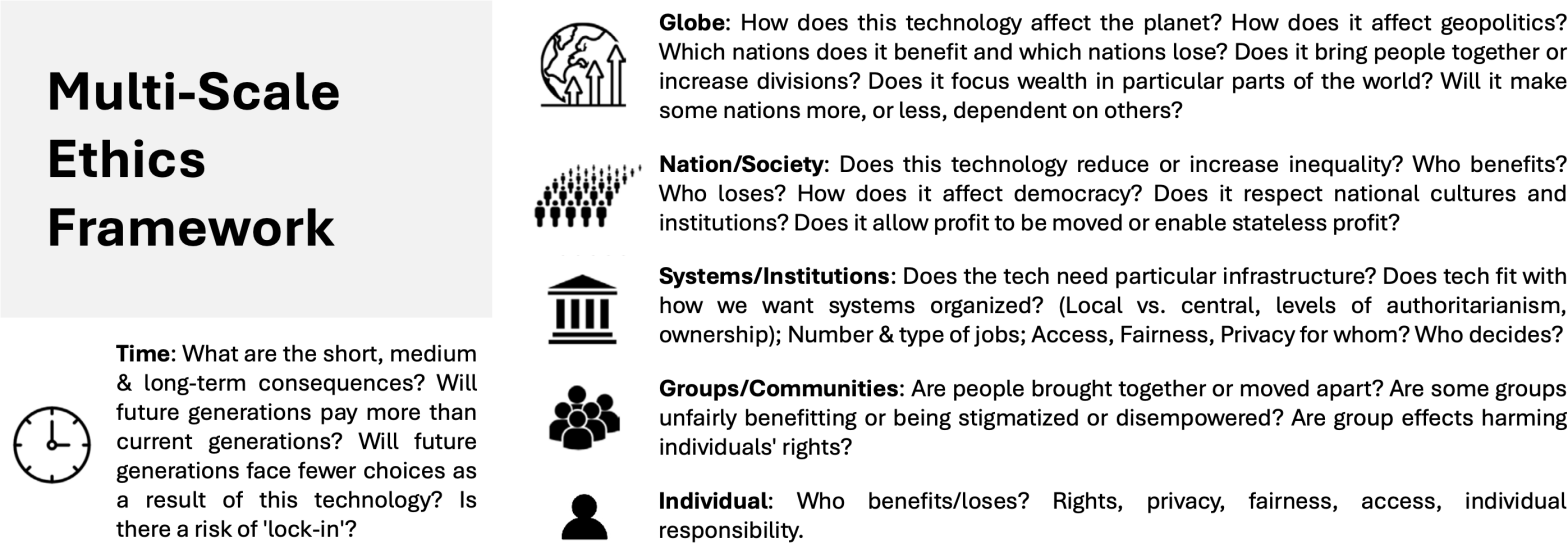
Multiscale Ethics Framework proposed to evaluate the ethical issues of AI at interactive levels of community. Reprinted from “Multi Scale Ethics—Why We Need to Consider the Ethics of AI in Healthcare at Different Scales,” M. Smallman, 2022. *Science and Engineering Ethics*, 28(6), 63.

This framework defines AI as a “socio-technical” system and highlights the lack of a structured approach to contextualizing its risks and benefits [38]. Most research focuses on ethical threats to individuals, such as privacy, autonomy, and transparency. However, risks also emerge at different levels and time scales beyond individual effects (Fig 1). Smallman suggests this framework can help identify risk patterns, anticipate recurring issues, and guide AI implementation while incorporating patient perspectives. Public forums and similar dialogs are essential for inclusive decision-making, ensuring ethical concerns are comprehensively addressed.

### “SHIFT” acronym for standardization

#### Sustainability, Human Centeredness, Inclusiveness, Fairness, Transparency (SHIFT).

**Fig 2:**
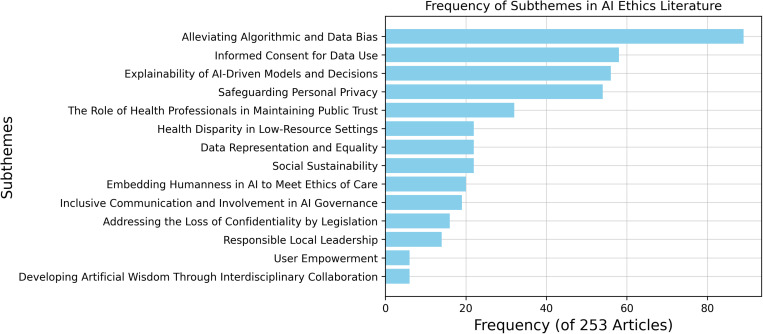
Frequency of key subthemes in AI ethics literature. Algorithmic bias, informed consent, explainability, and privacy emerge as the most prevalent concerns in responsible AI implementation in healthcare.

A thematic analysis of recent literature [39] identified key subthemes in AI ethics across 253 articles, with their corresponding frequencies: responsible local leadership (14), social sustainability (22), embedding humanness in AI (20), the role of health professionals in public trust (32), interdisciplinary collaboration for artificial wisdom (6), inclusive AI governance (19), mitigating algorithmic and data bias (89), data representation and equality (22), health disparities in low-resource settings (22), privacy protection (54), explainability of AI models (56), legislative safeguards for confidentiality (16), user empowerment (6), and informed consent for data use (58). Fig 2 illustrates the distribution of these concerns in responsible AI implementation in healthcare.

Standardized acronyms like “SHIFT” can help establish consensus on key AI challenges and protective initiatives for patients and communities. This work is crucial for educating stakeholders on AI applications in medicine. Siala and Wang highlight responsible initiatives, including linking algorithm outputs to human decision-making, implementing a centralized institutional review board, and integrating diverse patient data to enhance explainability [39].

### Responsible innovation focused on inclusion

Responsible healthcare AI innovation requires ethical, equitable technologies that prioritize patient needs while preventing harm, mitigating bias, and promoting inclusivity. Algorithmic bias, which can worsen healthcare inequities, remains a major challenge. Julia Trabulsi, a BioTech product lead and advisor, advocates for building meaningful controls, considering all users, and prioritizing people over profits. In a Dartmouth guest lecture on February 7, 2024, she emphasized oversampling underrepresented communities to balance data and reduce bias. Recognizing that data reflects societal biases, she stressed the need for inclusivity and fairness throughout AI development to ensure ethical implementation.

### Algorithmovigilance

Inspired by “pharmacovigilance,” this concept emphasizes continuous evaluation of AI algorithms to mitigate bias and ensure fairness. Bias can emerge at any stage of development due to factors like sample size, historical bias, representation bias, sponsorship bias, and self-serving bias [29]. Polevikov recommends best practices such as incorporating effect size and confidence intervals, using appropriate sample sizes, and ensuring transparency to avoid “data shopping.”

A key priority is ensuring AI enhances rather than disrupts healthcare and the provider–patient relationship. One potential solution is incorporating uncertainty measures into models, allowing providers to assess the reliability of AI-generated recommendations.

In the following discussion section, we will cover important takeaways from the current state of the field and the directions that need to be explored to ensure the use of AI in the medical field aligns with ethical standards.

## Discussion

### Is the development of healthcare AI fair and not biased?

The rapid advancement of AI and machine learning in healthcare presents significant challenges in maintaining ethical standards and regulatory oversight. Key concerns include fairness, transparency, consent, accountability, and equitable care, yet addressing these issues is difficult as understanding AI models often comes through their implementation. Bias remains one of the most pressing issues, particularly due to the lack of standardization in industry regulations and review processes.

Bias audits typically occur in early development phases, leaving later stages unchecked. Even FDA-cleared AI imaging products have shown problematic practices—only 64% of products from November 2021 used clinical data for validation, with just 4% reporting patient demographics, and 5% providing machine specifications. Only 34% of these models were validated by multiple institutions [40]. Bias often stems from mismatches between training populations and real-world clinical data. Studies indicate that Black, Hispanic, and female patients are less likely to receive CPR, regardless of income or location [41,42], leading to their underrepresentation in cardiac imaging datasets. This disparity affects AI model accuracy for disease prediction. NIH-funded initiatives like AIM-AHEAD and Bridge2AI have identified this misalignment as a major challenge in mitigating bias [40]. Some experts advocate for using datasheets or checklists to ensure datasets are representative and balanced.

Bias can arise at multiple stages of model development, including through exclusion, annotator subjectivity, funding sources, and objective mismatches. A lack of diversity in development teams further exacerbates these issues [40]. Encouraging collaboration among clinicians, analysts, and patient advocacy groups could help address these gaps. Some propose an oversight review before AI deployment in healthcare, where interdisciplinary experts assess bias, transparency, and ethical implications [40]. The risk of exacerbating bias remains a critical concern [39], underscoring the need for diverse representation in data collection, rigorous validation methods, and ongoing dialogue to refine AI models for equitable healthcare delivery.

### Is the deployment of healthcare AI patient-centered?

The American Medical Association (AMA) committed in 2023 to developing policies addressing unforeseen conflicts in AI-driven healthcare, acknowledging widely recognized ethical concerns [43]. However, AI ethics still lacks standardized protocols and a framework that considers its societal impact at multiple levels. Smallman’s “Multi-Scale Ethics” model highlights the need for a broader perspective on bias mitigation and responsible AI use. Strengthening protocols for responsible innovation and ensuring algorithmic bias monitoring, including balancing datasets to reduce disparities, are crucial steps toward ethical AI implementation.

Two key regulatory frameworks that balance innovation with safety and privacy are the European Union’s General Data Protection Regulation (GDPR) and the US Food and Drug Administration (FDA) oversight of AI-based medical devices [44]. The GDPR employs a risk-based approach, categorizing AI applications as unacceptable, high, or limited risk, with high-risk applications including medical devices and critical infrastructure. The FDA mandates pre-market evaluations for high-risk devices, ongoing monitoring, and strict quality controls. The Federal Trade Commission (FTC), alongside other U.S. agencies, has required firms to eliminate AI algorithms trained on improperly collected data [45]. It also oversees AI used in socioeconomic decisions, bias monitoring, and deceptive marketing claims [46]. The Equal Employment Opportunity Commission enforces anti-discrimination regulations in AI systems. The National Institute of Standards and Technology (NIST) is developing a US AI Bill of Rights covering safety, algorithmic discrimination, data privacy, informed consent, and human oversight. The US AI Executive Order on Safe, Secure, and Trustworthy Artificial Intelligence aligns with principles of fairness, transparency, and accountability.

Most regulatory frameworks are post-hoc, requiring resubmission processes that discourage rigorous ethical reviews at early stages [40]. Additionally, high validation costs, including dataset preparation, interdisciplinary expertise, and regulatory compliance, create barriers to responsible innovation. While necessary data protections safeguard confidentiality, they also restrict data availability, complicating the development of robust and unbiased AI models.

### Is the use of healthcare AI ethical? If not, how can policy be changed to ensure ethical implementation?

Despite advancements, no existing framework fully ensures seamless and ethical AI implementation in healthcare. A major challenge is the lack of interdisciplinary collaboration. Ethics is often perceived as a set of static guidelines, but it should be an ongoing process of moral decision-making. Organizations must engage in regular discussions on AI ethics, integrating input from healthcare ethicists, developers, researchers, and clinicians.

A disconnect persists between AI developers and ethicists due to the demands of engineering and development. Addressing this gap requires new initiatives that bring together diverse stakeholders, including patients, to discuss ethical concerns and promote equitable AI use. Beyond AI development, diversity should be reflected in research teams, advisory committees, and leadership positions. Partnerships with minority-serving institutions and community organizations can foster a more inclusive and innovative research environment.

There is a strong need for long-term studies on AI’s effects in healthcare, particularly regarding patient outcomes, efficiency, and best practices. Research on treatment efficacy, cost-effectiveness, patient satisfaction, and workflow impact is crucial [44]. The SHIFT framework proposed by Siala and Wang emphasizes not only ethical considerations but also the broader societal implications of AI [39]. Despite challenges, AI integration should be approached with optimism. Automation of repetitive tasks can allow healthcare professionals to focus more on patient care, while AI-driven diagnostics can enhance decision-making without replacing human expertise [44].
